# Targeting monoamine oxidase A: a strategy for inhibiting tumor growth with both immune checkpoint inhibitors and immune modulators

**DOI:** 10.1007/s00262-023-03622-0

**Published:** 2024-02-13

**Authors:** Yifan Ma, Hanmu Chen, Hui Li, Zhite Zhao, Qingling An, Changhong Shi

**Affiliations:** 1 Division of Cancer Biology, Laboratory Animal Center, Fourth Military Medical University, Xi’an, 710032 Shaanxi People’s Republic of China; 2 Gansu University of Traditional Chinese Medicine, Lanzhou, 730030 Gansu People’s Republic of China; 3 School of Basic Medical Sciences, Medical College of Yan’an University, 580 Bao-Ta Street, Yanan, 716000 Shaanxi People’s Republic of China; 4 Department of Urology, Xijing Hospital, Fourth Military Medical University, Xi’an, 710032 Shaanxi People’s Republic of China

**Keywords:** Monoamine oxidase A (MAOA), Neurology, CD8^+^ T cells, Tumor-associated macrophages (TAM), Immunotherapy

## Abstract

Monoamine oxidase A (MAOA) is a membrane-bound mitochondrial enzyme present in almost all vertebrate tissues that catalyzes the degradation of biogenic and dietary-derived monoamines. MAOA is known for regulating neurotransmitter metabolism and has been implicated in antitumor immune responses. In this review, we retrospect that MAOA inhibits the activities of various types of tumor-associated immune cells (such as CD8^+^ T cells and tumor-associated macrophages) by regulating their intracellular monoamines and metabolites. Developing novel MAOA inhibitor drugs and exploring multidrug combination strategies may enhance the efficacy of immune governance. Thus, MAOA may act as a novel immune checkpoint or immunomodulator by influencing the efficacy and effectiveness of immunotherapy. In conclusion, MAOA is a promising immune target that merits further in-depth exploration in preclinical and clinical settings.

**Supplementary Information:**

The online version contains supplementary material available at 10.1007/s00262-023-03622-0.

## Introduction

Monoamine oxidase A (MAOA) is located on the outer mitochondrial membrane and catalyzes the degradation of biogenic and dietary-derived monoamines. MAOA serves functional roles in the brain by regulating the dynamic balance of key monoamine neurotransmitters, including catecholamines (such as dopamine, norepinephrine, and epinephrine) and indoleamines (mainly serotonin). MAOA influences the emotional and behavioral states of people through the abovementioned neurotransmitters [1, 2]. The results of genetic correlation studies have identified several MAOA gene variants associated with altered MAOA expression levels: Low-activity forms of the MAOA gene are related to aggression and hyperactivity disorders, whereas high-activity forms are associated with depressive disorders [3, 4]. Thus, monoamine oxidase A inhibitors (MAOAIs) have been developed against active MAOA variants and used clinically to treat the symptoms of depression [5]. MAOA is involved in tumorigenesis, cardiovascular diseases, diabetes, and obesity in addition to its importance in brain function [6–8]. MAOAIs multifaceted nature may be an advantage, making them suitable for treating several disorders.

Neurons and immune cells share a wide range of signal transduction pathways, cell surface receptors, and secretory molecules [9]. Immune cells activated by the common biochemical language of the nervous and immune systems synthesize a wide range of neurotransmitters, neuropeptides, hormones and their receptors, and cytokines [10]. A variety of neurotransmitters such as serotonin, dopamine, and norepinephrine which are dynamically regulated by MAOA modulate immune responses. Macrophages, dendritic cell (DC), T cells, and other immune cells express ionotropic, metabotropic, and G-protein-coupled receptors for various neurotransmitters [11]. Increasing evidence indicates that neurotransmitters and neuropeptides previously thought to be neuron-specific are also expressed in immune cells, including the X-linked recessive gene MAOA, and their functions in the immune response are gradually being revealed [12]. In this review, we summarize the ways that MAOA helps regulate tumor immunotherapy through various types of immune cells (such as CD8^+^ T cells and tumor-associated macrophages), and conclude by arguing that MAOA may act as a novel immune checkpoint or immunomodulator that regulates tumor-associated immune cell metabolism, thereby influencing the efficacy and effectiveness of immunotherapy. Exploring the application of MAOAIs or combining MAOAIs with existing cancer immunotherapy, chemotherapy, and molecularly targeted therapies is particularly important for improving the prognosis of patients with tumors and prolong their survival rates.

## The neurological origin of MAOA influences its role in tumor immunization

Millions of years of evolutionary pressures and processes have shaped the co-evolution of mammalian neurology and immunity to co-maintain dynamic cellular and physiological homeostasis in the context of changes occurring within and outside an organism (e.g., blood pressure, pH, or temperature) [9]. Jerne et al. [13] were among the early researchers to suggest functional similarities between the nervous and immune systems in terms of their recognition mechanisms and memory formation capabilities. These similarities are evident in their evolutionary adaptation to changing external conditions, sensing, integration, and responses to injuries originating from outside the environment or inside the body at their different interfaces (ports), including damaged, infected, and malignant tissues [10]. The nervous system has its own specific tissue and cellular morphological structures, whereas the immune system consists of mobile and dispersed cells; it was evolutionarily meaningful to preserve some similar molecular regulatory pathways between both defense systems.

Neurons can synthesize and secrete various cytokines (such as IL-6) [14, 15]. In response, diverse neurogenic transmitters (such as norepinephrine) and their binding sites are produced by immune cells [16]. These common shared mediators provide a material basis for information exchange between the nervous and immune systems. Similarly, MAOA plays key roles in regulating neurodegeneration, neuron death, and neuron activity in neuronal synapses and affects T cell reactivity through “immune synapses” via immunosuppressive tumor-associated macrophages (TAMs) and tumor growth [17, 18]. MAOA regulates neurotransmitter homeostasis to engage with synaptic transmission processes in the central nervous system, which in turn affects central neuronal reactivity and peripheral neurophysiological functions [19]. MAOA catalyzes the oxidative deamination of several monoamine neurotransmitters—including serotonin, norepinephrine and dopamine into aldehydes—with the synthesis of ammonia and hydrogen peroxide as by-products [20]. The abnormally elevated levels of MAOA activity result in increased production of harmful by-products: hydrogen peroxide (H_2_O_2_) and aldehydes [21]. The excessive by-products lead to cellular oxidative stress that causes mitochondrial toxicity linked to aberrant cellular signaling which may lead to cancer development [22]. Additionally, MAOA enzyme causes deoxyribonucleic acid (DNA) damage and oxidative cell injury [23]. It may be involved in tumorigenesis via reactive oxygen species (ROS) production from oxidative deamination reactions. The functions of MAOA in immune responses are gradually becoming revealed. MAOA can directly influence the expression of immunosuppressive molecules (immune checkpoints) on immune cells (to subsequently regulate immune cell activation) or regulate the tumor microenvironment (TME) by affecting tumor-associated immune cell metabolites [17, 18].

Immunosuppressive properties are commonly considered dominant in the TME and cannot be ignored in terms of malignant tumor progression, immune escape, and treatment resistance. To overcome this suppression and harness the antitumor potential of immunocytes, several immune checkpoint blockade (ICB) therapies have been developed over the past few years [24, 25]. Blocking the cytotoxic T lymphocyte-associated protein 4 (CTLA-4)–programmed cell death protein 1 (PD-1)–programmed cell death ligand 1 (PD-L1) inhibitory pathway has enabled remarkable clinical responses and revolutionized the treatments of different cancers [26–28]. However, only a small number of patients benefit from ICB therapy due to individual differences among patients, immune-related adverse events [29], and the development of secondary drug resistance [30]. Additional molecular targets are urgently needed to expand the family of immune checkpoints, highlighting the need for exploring the functions of existing checkpoints during multiple phases and considering different perspectives of the immune response, such as cellular metabolic processes occurring in immune cells. MAOA, a classical molecule of neurological origin, participates in regulating the dynamic balance of metabolites in tumor-associated immune cells and is expected to join the immune checkpoint family, which provides a rationale for targeting MAOA to ameliorate immune metabolic disorders associated with the TME.

## Roles of MAOA in CD8^+^ T cells

ICB is most often applied to treat solid tumors with the goal of overcoming inhibitory signals and activating endogenous antitumor-specific T cells. CD8^+^ T cells play key roles in adaptive immunity against cancer, and their “metabolism” (e.g., recognition reply, regulatory killing, and effector functions) strongly influences the entire TME. CD8^+^ T cells are not always highly responsive to tumor antigens. When unable to produce effector cytokines and cytotoxic molecules (e.g., granzyme and perforin), CD8^+^ T cells are typically characterized by terms such as “incompetent,” “tolerant,” or “exhausted” to indicate low responsivity. Potential factors contributing to CD8^+^ T cell dysfunction include impaired T cells infiltration to tumor sites, a state of exhausted differentiation due to high expression of inhibitory receptors, and functional disabilities due to alterations in epigenetic markers. Previous findings showed that MAOA was expressed as a key molecule in tumor-infiltrating T cells (TILs), resulting in T cell dysfunction. In particular, MAOA was significantly upregulated in exhausted CD8^+^ T cells [17]. Furthermore, TIMER databases (http://cistrome.org/TIMER/) were used to determine the relationship between MAOA expression and immune infiltration. The results of bioinformatics analysis also demonstrated a significant negative correlation between the expression of MAOA and the immune infiltration of CD8^+^ T cells in melanoma and lung adenocarcinoma (Fig. S1A and 1C).

To investigate the correlation between the expression of the analyzed MAOA and the level of tumor-associated immune cell recruitment, the Tumor Immune Estimation Resource (TIMER) 2.0 portal was used (http://timer.cistrome.org/(accessed on 30 October 2023)). One such algorithm in TIMER 2.0 is the CIBERSORT algorithm, which allows for the analysis of 22 immune cell types, including CD8^+^ T cell and M2-type macrophage. Then, the immune component's “Gene Module” on TIMER 2.0 platform was used to retrieval. Thus, the correlation between the expression of MAOA in melanoma/lung adenocarcinoma and the abundance of CD8^+^ T infiltration/M2-type macrophage was obtained. The statistical significance of the correlation was estimated on the TIMER 2.0 platform using Spearman’s rank correlation coefficient. Results with *p* < 0.05 were considered statistically significant. Raw data are available in supplementary information—Table 01, Table 02.

### High MAOA expression in exhausted CD8^+^ T cells

Exhausted T (Tex) cells belong to a unique cell lineage, consisting mainly of heterogeneous cells, including progenitor and terminal T cell subsets [31]. In contrast to memory T cells and effector T cells, Tex cells are characterized by a progressive loss of effector functions, high and sustained expression of inhibitory receptors, metabolic dysregulation, poor memory recall and homeostatic self-renewal, and distinct transcriptional and epigenetic programs. Inhibitory receptors function as crucial negative regulatory pathways that control autoreactivity and immunopathology. Although inhibitory receptors are transiently expressed in functional effector T cells during activation, higher and sustained expression of inhibitory receptors is a hallmark of Tex cells. Tex cells consistently express high levels of PD-1, and blocking PD-1 improves or somewhat delays T cell exhaustion and induces potent killing of cancer cells by immune cells [32]. Additionally, as negative regulatory immune molecules, both T cell immunoglobulin and mucin domain-containing molecule 3 (Tim-3) and lymphocyte activation gene 3 (LAG-3) are expressed in dendritic cells (DCs), CD4^+^ T cells, CD8^+^ T cells, and several other types of cells [33, 34]. Similarly, Tex cells also are characterized by high expression of inhibitory receptors, which naturally include Tim-3 and LAG-3, both of which bind to cognate ligand to suppress effector T cell activity and promote immune tolerance in the TME. Typically, the higher the number of inhibitory receptors co-expressed by Tex cells, the more severe the exhaustion. These co-expression patterns are mechanistically relevant, as simultaneously blocking multiple inhibitory receptors synergistically reverses T cell exhaustion [32].

The MAOA gene was readily induced in tumor-infiltrating CD8^+^ T cells, and MAOA expression levels positively correlated with T cell exhaustion and a dysfunctional status [18]. Cells co-expressing Tim-3 and LAG-3 with the highest levels of MAOA mRNA were considered the “most exhausted” PD-1^hi^ cells. That is, the MAOA gene was highly expressed in the most “exhausted” tumor-infiltrating CD8^+^ T cells (PD-1^hi^Tim-3^hi^LAG-3^hi^). T cell exhaustion is non-redundantly regulated by various inhibitory pathways, and simultaneous blockade of the T cell inhibitory receptors PD-1 and LAG-3 synergistically improves T cell responses and delays disease progression. In a similar way, the synergistic blockade of PD-1 and TIM-3 can also co-regulate Tex cells. From another perspective, cells with greater PD-1 depletion that co-express Tim-3 and LAG-3 may benefit the most from MAOAI treatment [32, 34].

Tumors have evolved different ways of limiting human leukocyte antigen-1 (HLA-1)-dependent antigen presentation and immune evasion; thus, tumor-antigen recognition during antitumor therapy cannot be overlooked. MAOA inhibits the response of CD8^+^ T cells to antigenic stimulation, and MAOA expression is induced by antigenic T cell receptor stimulation of CD8^+^ T cells, which in turn inhibits T cell activation. This negative feedback loop reveals MAOA as a new immune checkpoint that functions as a negative feedback regulator induced by tumor antigen recognition that suppresses the antitumor responsiveness of CD8^+^ T cells. Thus, MAOA has joined the expanding family of immune checkpoints including PD-1/PD-L1, CTLA-4, TIM-3, LAG-3, T cell immunoreceptor with Ig and ITIM domains, V-domain Ig suppressor of T cell activation, and others [35].

### MAOA regulates serotonin metabolism in T cells

**Fig. 1:**
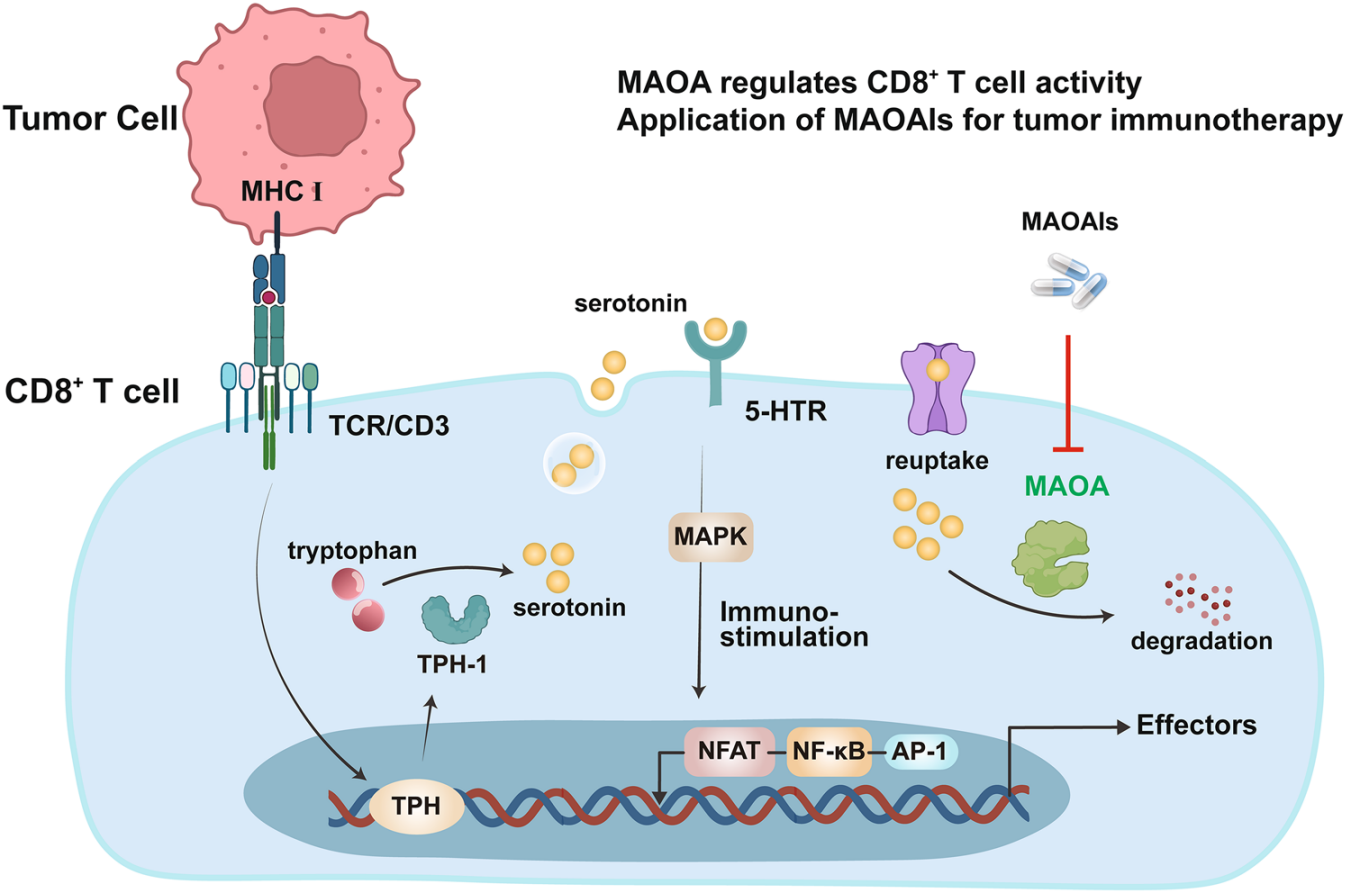
Mechanism whereby MAOA regulates CD8^+^ T cell activity and the application of MAOAIs in tumor immunotherapy

CD8^+^ T cells can synthesize serotonin, which has been implicated as an accessory signal that enhances T cell activation by signaling through T cell surface serotonin receptors (5-hydroxytryptamine receptors, 5-HTRs) (Fig. 1) [36, 37]. Prior findings indicated that MAOA helped regulate the antitumor immunity of CD8^+^ T cells by participating in “an antigen stimulation-induced serotonin synthesis/degradation loop in CD8^+^ T cells” [18]. MAOA knockout (KO) mice exhibited significant tumor growth inhibition in two homozygous mouse tumor models. This improved tumor-suppressive response was accompanied by increased release of the cytokine IL-2 and the cytotoxic molecule interferon-gamma in MAOA KO mice, suggesting that knocking out MAOA led to increased CD8^+^ T cell activity.

CD8^+^ T cells derived from MAOA wild-type (WT) and MAOA KO mice were cultured in vitro, and treatment with an anti-CD3 antibody mimicked antigenic stimulation. CD8^+^ T cells from MAOA WT mice showed upregulated expression of TPH1, a rate-limiting enzyme that controls serotonin synthesis, and MAOA induce serotonin degradation, suggesting the presence of a CD8^+^ T cell antigen stimulation-induced synthesis/degradation loop for serotonin. In functional terms, MAOA deficiency did not interfere with serotonin synthesis, but instead tended to impede serotonin degradation, leading to increased serotonin secretion by CD8^+^ T cells [18]. Comparing the molecular signaling pathways of CD8^+^ T cells from MAOA WT and MAOA KO mice after antigen stimulation revealed enhanced signaling through key pathways in T cells from MAOA WT mice. These pathways included the mitogen-activated protein kinase (MAPK) signaling pathway, such as increased extracellular signal-regulated kinase (ERK) phosphorylation and signaling downstream of the T cell receptor, including nuclear translocation of nuclear factor of activated T cells (NFAT), nuclear factor-κB (NF-κB), and c-Jun transcription factors. Blocking 5-HTRs substantially inhibited these enhancements. The authors also showed that serotonin signaling in mouse CD8^+^ T cells may have been mainly mediated through Htr2b and Htr7; in particular, Htr7 can mediate activation-related signaling events (Fig. 1) [18, 38]. These findings suggest CD8^+^ T cells from MAOA KO mice produced higher levels of serotonin, which acted as an autocrine immunomodulator and activated the downstream immunostimulatory mitogen-activated protein kinase pathway, in turn promoting “cross talk” with the T cell receptor (TCR) signaling pathway, leading to the enhanced effector function observed in CD8^+^ T cells from MAOA KO mice(Fig. 1).

Several reports elucidated multiple links between serotonin and cancer progression [39, 40]. Acting as an acute inflammatory agent and chemoattractant [41, 42], serotonin influences various immunological functions and can promote anti-inflammatory macrophage polarization, an effect that is potentially relevant in tumor growth [43]. Schneider et al. [44] found that genetic knockdown of peripheral serotonin enhanced CD8^+^ T cell accumulation in tumors and reduced tumor growth using syngeneic mouse models of pancreatic and colorectal cancer. In addition, the pharmacological serotonin inhibitors, fluoxetine and telotristat, enhanced the effects of anti-PD-1 therapy to induce long-term tumor control in mice. Both fluoxetine (a selective serotonin reuptake inhibitor, SSRIs) and MAOAIs were applied in that study, and the latter were used as first-line drugs for treating depression, supporting the idea that certain antidepressants may have antitumor benefits. Regarding T cell activation, lower serotonin concentrations may augment T cell activation [45], whereas higher serotonin concentrations display an inhibitory effect [46]. The extent of T cell activation may also be related to the source of serotonin; thus, autocrine stimulation with serotonin secreted by T cells and paracrine stimulation with serotonin from the peripheral circulation may have distinct effects on T cell activation.

Indeed, MAOA-mediated oxidative deamination of serotonin generated large amounts of H_2_O_2_ and reactive oxygen species (ROS), and low levels of ROS played a key role in antigen-specific T cell activation [47]. In contrast, high levels of ROS were toxic to lymphocytes, particularly TAMs. These data suggest the importance of exploring the functions of MAOA in other types of immune cells associated with different metabolic processes in future studies.

## Roles of MAOA in TAMs

TAMs are key components of an immunosuppressive TME that suppress T cell antitumor responsiveness in most solid tumors and play roles in tumor angiogenesis, extracellular matrix remodeling, cancer cell proliferation, metastasis, and resistance to chemotherapeutic agents and checkpoint blockade therapy [35, 48]. TAMs belong to the macrophage family, are highly plastic, and can activate different types of effector functions in response to different stimuli [49, 50]. Tumor promotion and immunosuppressive polarization are dominant characteristics of TAMs. Cancer treatment strategies targeting TAMs can be broadly classified into two categories, namely (1) strategies that deplete TAMs and (2) strategies that alter the immunosuppressive activity of TAMs [51, 52]. However, an inherent disadvantage of depleting TAMs is the loss of their natural immunostimulatory role as major phagocytes and specialized antigen-presenting cells (APCs) in solid tumors. Correspondingly, when properly induced or activated, TAMs can mediate phagocytosis and cytotoxic killing against cancer cells, engaging in bidirectional interactions with innate and adaptive immune cells. Given the high degree of plasticity of TAMs, reprogramming immunosuppressive polarization is an essential strategy for developing future tumor immunotherapy with TAMs. The findings of one study identified MAOA as a critical regulator of TAMs and supported repurposing MAOAIs for reprogramming TAMs to improve cancer immunotherapy [17]. MAOA also helped regulate the T cell functions of TAMs in three-dimensional-culture experiments designed to ensure that key immune features of the natural TME were preserved, suggesting that MAOA-targeted TAM immunotherapy can modulate tumor immune responses from multiple angles [53]. Furthermore, the results of bioinformatics analysis also demonstrated a significant positive correlation between the expression of MAOA and the immune infiltration of M2 macrophages in melanoma and lung adenocarcinoma (Fig. S1B and 1D).

### Targeting MAOA Regulates/Reprograms TAM Polarization

**Fig. 2:**
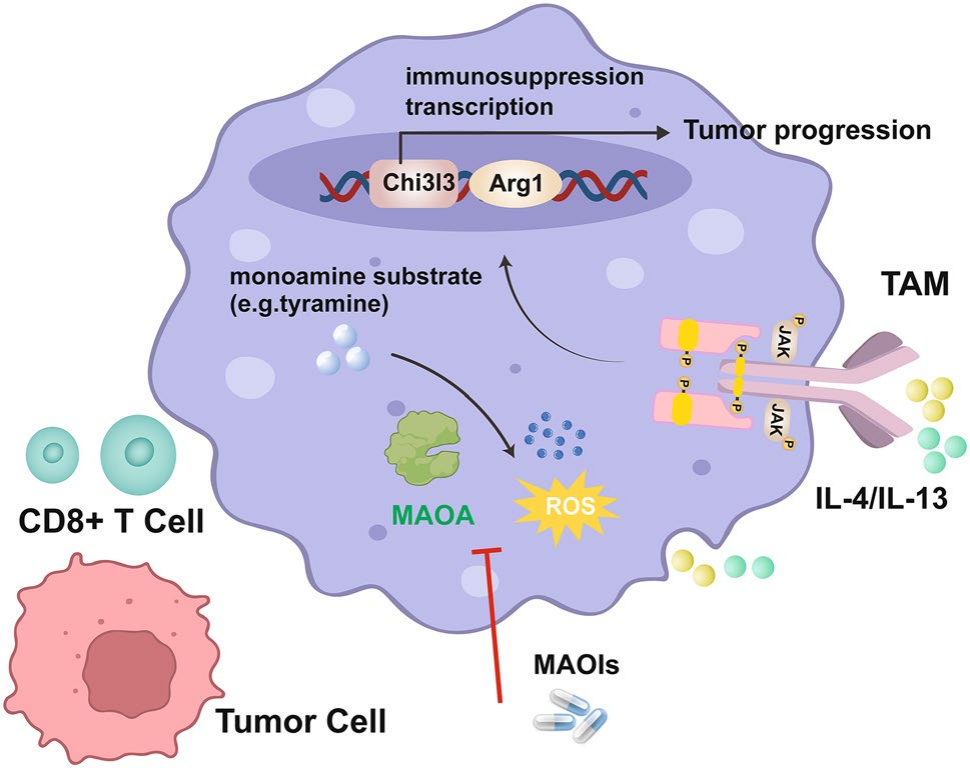
Mechanism whereby MAOA suppresses immunity and enables tumor progression and the application of MAOAIs for reprogramming TAM polarization and tumor immunotherapy

MAOA can promote macrophage polarization to an immunosuppressive phenotype (Fig. 2). By upregulating oxidative stress, MAOA promoted the immunosuppressive polarization of TAMs and suppressed their antitumor immunity in mice [17]. MAOAIs such as phenelzine (PLZ)-induced TAM reprogramming and inhibited tumor growth. MAOA acted as an autoregulatory factor that directly affected the direction of TAM polarization, thereby affecting the antitumor activity of T cells and tumor growth. During macrophage colony-stimulating factor (M-CSF)-induced macrophage differentiation, MAOA KO macrophages exhibited less of an immunosuppressive phenotype in response to IL-4/IL-13 stimulation than MAOA WT macrophages [17].

MAOA regulates the dynamic balance of monoamine transmitters in vivo by catalyzing the oxidative deamination of monoamines, which generates hydrogen peroxide (related to oxidative stress) as a by-product, thereby increasing intracellular ROS levels [54]. Another report showed that intracellular ROS (also related to oxidative stress) may activate the immunosuppressive characteristics of macrophages [55]. Both supplementation with tyramine, an MAOA substrate, and H_2_O_2_ increased ROS levels and upregulated the expression of immunosuppressive genes (i.e., Chi3l3 and Arg1) in MAOA WT (but not MAOA KO) bone marrow-derived macrophages. These findings suggest that MAOA regulates the immunosuppressive polarization of macrophages by modulating intracellular ROS levels. ROS can promote Janus kinase (JAK) and signal transducer and activator of transcription 6 (Stat6) phosphorylation in various types of cells [56, 57]. Indeed, direct analysis of TAMs isolated from MAOA WT and MAOA KO mice carrying B16-OVA tumors confirmed that MAOA impacted macrophage polarization by regulating ROS levels and thereby sensitizing the JAK-Stat6 signaling pathway. After IL-4/IL-13 stimulation, JAK is phosphorylated and subsequently phosphorylates Stat6; phosphorylated Stat6 dimerizes and migrates to the nucleus, where it binds to the promoters of IL-4- and IL-13-responsive genes including those involved in macrophage immunosuppressive functions (e.g., Chi3l3 and Arg1; Fig. 2) [58]. Collectively, these data suggest that MAOA promotes the immunosuppressive polarization of TAMs in the TME by upregulating intracellular ROS levels in TAMs and thereby enhancing the IL-4/IL-13-induced JAK–Stat6 signaling.

### Targeting MAOA Reverses TAM Inhibition During T Cell-Mediated Antitumor Activities

APCs with a suppressive phenotype (mainly TAMs and tolerant DCs) cannot elicit lymphocyte immune responses or induce lymphocyte exhaustion, both of which play crucial mechanistic roles in immune evasion by tumor cells [59, 60]. APCs with suppressive epitopes express inhibitory molecules on their cell surfaces and secrete extracellular matrix components, cytokines, chemokines, proteases, and metabolites to influence the effector functions of non-specialized APCs (including vascular endothelial cells, epithelial cells, stromal cells, fibroblasts, and activated T cells), which help establish a hostile and immunosuppressive TME. TAMs can interfere with effector T cell metabolism by releasing cytokines (such as IL-10 and TGF-β) and promoting autoimmune suppression of polarization during tumor progression [61]. TAMs can also suppress T cell-mediated antitumor immunity by upregulating immune checkpoint ligands, such as PD-L1 and PD-L2 [62]. An ex vivo three-dimensional culture model designed for TME mimicry comprised three major components of a human TME, including human tumor cells, TAMs, and tumor antigen-specific T cells. In this culture system, MAOA inhibition antagonized TAM-dependent suppression of T cell antitumor reactivity [53].

Further study revealed that the MAOA inhibitor PLZ significantly inhibited IL-4/IL-13-induced immunosuppressive polarization of monocyte-derived macrophage (MDMs) and significantly downregulated immunosuppressive markers (i.e., CD206) and immune checkpoint receptor ligands (PD-L1 and PD-L2) [17]. Mixed-macrophage/T cell in vitro response assays demonstrated that MAOA blockers effectively antagonized the immunosuppressive function of M2 macrophages. Peripheral blood mononuclear cell-derived T cells co-cultured with PLZ-treated M2 macrophages expanded faster than those co-cultured with untreated M2 macrophages and secreted more pro-inflammatory cytokines (i.e., IFN-γ and TNF-α), indicating that T cell expansion and activation were rescued. These findings also reflect the potential promise of MAOA blockade for TME-targeted cancer immunotherapy. Further experiments were performed using PLZ-treated macrophages to establish an 3D TME mock culture in vitro. Human tumor cells were then added to the PLZ-treated macrophages and mesothelin CAR-engineered T (MCAR-T) cell culture system to achieve co-culture of all three components. The activation and cytotoxicity of mesothelin CAR-engineered T cells were enhanced when co-cultured with PLZ-treated macrophages, as evidenced by granzyme B and CD25 upregulation and CD62L downregulation. MAOA blockade reprogrammed TAM polarization, which antagonized TAM immunosuppression and enhanced T cell antitumor responses [53].

## Prospects and expectations

Recently, researchers have identified numerous factors that influence the efficacy of tumor immunotherapy and promising new immune checkpoints or targets for tumor therapy abound. MAOA is a well-established target in the nervous system that has approved drugs (with known safety profiles and modes of action) that rapidly entered the clinic and demonstrated a rapid clinical translation of previous discoveries [63]. Many MAOAIs have been used to treat depression, Parkinson’s disease, and Alzheimer’s disease [64]. However, the selectivity differences between MAOAIs, the toxicity/side effects (tyramine reaction and hypertensive crisis) [65], and certain improvements (drug composition, dosage, or administration) must be determined for MAOAIs. Particularly, tumor immunotherapy focused on the suppressive TME may bring new and significant therapeutic benefits when combined with existing cancer therapies. The discovery of MAOA expression on other types of immune cells is also significant and may provide new perspectives for future study. A subset of solid tumors (e.g., prostate cancer, PCa) [66] co-express/co-localize MAOA within the TME, which comprises various cell types such as tumor cells, immune cells, and mesenchymal cells. Such co-expression should help maximize the benefits MAOAI treatment [67, 68], making it an ideal strategy for future synergistic therapy. Thus, future research involving MAOA as a new immunologically relevant target should consider or focus on several factors, as summarized below.

### Applications of MAOAIs

MAOAIs are defined by their pharmacological activities and are subclassified as irreversible and reversible MAOAIs [64]. In most peripheral tissues, the predominant MAO isozyme is MAOA [69]. The early MAOAIs inhibited MAOA irreversibly. When they interacted with MAOA, they permanently deactivated it, and the enzyme function was not restored until the enzyme is replaced. Newer MAOAIs, such as moclobemide, are reversible, meaning that when the inhibitor dissociates from the enzyme, the activity is restored. MAOAIs are also defined by their selectivity [64]. Some inhibitors selectivity inhibit isozyme A (e.g., moclobemide and clorgyline), whereas others are non-selective (e.g., PLZ and tranylcypromine), inhibiting both the A and B isozymes [70].

The efficacies of MAOAIs in different classes (including PLZ, clorgyline, and moclobemide) have been studied previously. Those MAOAIs efficiently induced CD8^+^ T cell hyperactivation, based on the observations of CD25, granzyme B, IL-2, and interferon-gamma upregulation. In a model of B16-OVA melanoma prevention, the MAOAIs markedly suppressed tumor growth [18]. Diverse MAOAIs, which were previous used or are currently being used extensively in clinical treatment, offer further possibilities for selecting drugs for repurposing in tumor immunotherapy.

The prevalence of major depression is four times higher in patients with cancer than in the general population, and up to one quarter of patients with cancer have clinically significant symptoms of depression and anxiety [71]. Therefore, repurposing MAOAIs for cancer immunotherapy may provide both antidepressant and antitumor benefits for patients. It is noted that bioinformatics analysis showed that, holistically, MAOA expression was negatively correlated with CD8^+^ T cell immune infiltration in most tumor types, however, there were also some tumor types that showed a positive correlation. This suggests that we should consider the optimal treatment strategy according to specific cancer types when applying MAOAIs for immunotherapy.

### Strategies for Improving MAOAIs

Earlier hydrazine-based MAOAIs were associated with hepatotoxicity and were withdrawn from clinical use [72]. Irreversible MAOA inhibition can affect the intestinal metabolism of tyramine, leading to a potentially fatal hypertensive crisis when patients ingest tyramine-rich foods such as cheese [73, 74]. When MAOA is inhibited, the capacity to handle tyramine intake from the diet decreases significantly. This causes the brain to become vulnerable to overstimulation by postsynaptic adrenergic receptors after ingesting a small dose of tyramine (8–10 mg), which in turn can result in life-threatening blood pressure elevations [75]. Therefore, drugs that irreversibly inhibit MAOA (e.g., tranylcypromine) have dietary restrictions, making them less appealing candidates for treating neurological disorders and as ICB therapies than other available options. Even reversible MAOAIs (e.g., moclobemide) can partially alleviate the abovementioned side effects, although they cannot entirely eliminate them [65]. To fundamentally resolve hypertensive crises and dietary restrictions, the effects of MAOAIs on intestinal tyramine metabolism need to be further addressed. Alternative administration routes outside of the intestines (e.g., transdermal drug delivery) [76] or modified and improved drug formulations are expected to provide new application scenarios for MAOAIs [77, 78].

When administered at high doses or in combination with other serotonin analogs, MAOAIs have been associated with serotonin syndrome, which results from the overstimulation of serotonin receptors and causes adverse neurological and physical responses such as delirium and neuromuscular hyperactivity [5]. In addition, MAOAIs (as a class of antidepressants repurposed for cancer treatment) may induce aggressive behavioral side effects when administered at immunotherapeutic doses [3]. Previous data demonstrated that cross-linked multilamellar liposomal vesicles containing the MAOI PLZ could be used to avoid these neurological side effects while simultaneously improving antitumor activity. The results of that study also showed that cross-linked multilamellar liposomal vesicle-PLZ treatment had greater antitumor efficacy in a B16-OVA mouse model of melanoma than treatment with free PLZ [79]. The authors also demonstrated that nanoformulating PLZ resulted in the complete elimination of MAOI-related aggression. Developing nanoformulated PLZ for cancer therapy is a novel therapeutic strategy for repurposing MAOAIs as ICB therapeutics.

### New combination therapy strategies

Multiple cancer treatment modalities can be combined with MAOIs. For example, MAOI significantly synergized with anti-PD-1 to suppress tumor growth in mice, and MAOA expression levels dictated the survival of patients with melanoma who received anti-PD-1 therapy [18]. These findings highlight the need for combination therapies. MAOA inhibitor has been applied to multiple synergistic therapeutic strategies: Simon K P Schmich et al. [80] proposed MAOA expression with pancreatic ductal adenocarcinoma-related muscle wasting and the therapeutic potential of the MAOA inhibition with harmine hydrochloride. Chen jin et al. [81] synthesized doxorubicin (DOX) and isoniazid (INH, a MAOA inhibitor) conjugates through a pH sensitive hydrazone bond. Results demonstrated that DOX-INH could effectively enhance the activity of CD8^+^ T cells and perform a synergistic anti-tumor effect with PD-L1 small molecular inhibitor. Jessica A. Lapierre et al. [82] reported that the deletion of MAOA delayed prostate tumor development in the MAOA/Pten DKO mouse model of prostate adenocarcinoma, and high lever markers of immune stimulation (such as CD8^+^ cytotoxic T cells, granzyme B, and IFN-γ) have been detected in this DKO mice while decreasing expression of markers of immune suppression (such as FoxP3, CD11b, HIF-1-alpha, and arginase 1) were found. Conventional chemo-/radiotherapies and newer immunotherapies such as ICB therapy for patients with cancer often induce or exacerbate depressive symptoms. These central nervous system-related side effects are thought to be related to treatment-induced immune responses and inflammation [83]. Therefore, combining MAOIs (with antidepressant properties) in cancer therapy may both improve both the antitumor efficacy and mitigate central nervous system side effects.

Syngeneic mouse tumor model studies (i.e., the B16-OVA melanoma and MC38 colon cancer models) provide proof-of-principle evidence for the cancer immunotherapeutic potential of MAOIs, especially when used in combination with ICB therapies such as PD-1/PD-L1 blocking [18]. With both the immunotherapy-sensitive MC38 colon cancer model and the less immunotherapy-sensitive B16-OVA melanoma model, a MAOAI (PLZ) effectively inhibited tumor growth similarly to anti-PD-1 therapy, and subsequent combinations of PLZ and anti-PD-1 therapy produced better efficacy and completely inhibited tumor growth. It is reasonable to believe that the tumor-suppressive effects of PLZ were likely mediated by its immunomodulatory function.

### Regulation of other types of tumor-associated immune cells

The immunomodulatory function of MAOA is certainly not limited to the regulation of CD8^+^ T cells and TAMs. Some investigators have detected MAOA expression in other types of immune cells (e.g., DCs, macrophages, and regulatory T [Treg] cells) [18]. In MAOA KO mice, high responsiveness has also been observed with multiple types of immune cells. The innate immune response is an important regulator of tumor growth, where macrophages serve a critical link. A previous report showed that clorgyline delayed glioma progression by increasing macrophage infiltration into tumors in situ [84]. Significant increases in the numbers of macrophages and TNF-α positive cells among tumors in MAOAI-treated animals were detected when compared to those in untreated animals, suggesting that MAOAIs upregulate pro-inflammatory responses and reduce tumor progression.

DCs are known as the most potent type of APCs and are best characterized by their ability to stimulate the activation and proliferation of initial T cells (the initiators of specific immune responses) [85, 86]. MAOA expression has been observed in DCs [18], similar to macrophages, and targeting MAOA in DCs for immunomodulation or metabolic regulation may provide new directions and possibilities for future tumor immunotherapy. In solid tumors, beside TAMs, Treg cells have been detected in immunosuppressive TMEs, and MAOA may serve as a new target in the future, perhaps improving the immunosuppression and immune incompetence of Tregs.

### Combination immune therapy for PCa

MAOA is highly expressed in PCa [87], and its degree of expression varies with disease development [88]. Previous results demonstrated that MAOA was associated with PCa progression and played key roles during almost every stage of PCa development, including castrate-resistant PCa, neuroendocrine PCa, metastasis, drug resistance, stemness, and perineural invasion. Mounting evidence has suggested that MAOA expression affects the proliferation and metastasis of PCa. Moreover, MAOA is not only expressed in PCa cells but also in other types of epithelial cells, stromal cells, intratumoral T cells, and TAMs, which collectively enhance cancer progression and metastasis [87, 89]. Cancer-associated fibroblasts (CAFs) are the most abundant and important type of stromal cells in the PCa TME, and MAOA expression in CAFs increases in parallel with cancer progression [90]. Furthermore, targeting MAOA can disrupt its cross talk with the androgen receptor to restore enzalutamide sensitivity, block glucocorticoid receptor activity, and androgen receptor-dependent PCa cell growth, suggesting it as a potential strategy for immune checkpoint inhibition [91] to alleviate immune suppression and enhance T cell immunity-based cancer immunotherapy.

MAOAIs can directly inhibit the growth of PCa cells resistant to anti-androgen therapy, possibly by regulating autophagy and apoptosis in cancer cells, suggesting that MAOAIs may target and inhibit the progression of some cancers through different mechanisms, leading to tumor therapy with a single molecule through a multitarget/multimechanism pathway [92, 93]. Therefore, targeting MAOA is a promising emerging strategy for the integrated treatment of PCa, involving multiple sites, perspectives, and pathways.

## Conclusion

MAOA has long been studied as a neurotransmitter and is now known to be expressed on multiple types of immune cells in the TME. As a new immune checkpoint, MAOA characterizes the degree of T cell exhaustion and stimulates immunosuppression polarization in TAMs. As an immunomodulator, MAOA targets cellular metabolites and not only regulates CD8^+^ T cell reactivity from its own perspective, but also promotes TAM immunosuppression to reduce T cell antitumor responses. Therefore, targeting MAOA is a promising immunotherapy strategy with the combined benefits of restoring T cell antitumor effects and reprogramming TAM polarization. In the future, improving the compositions of MAOAIs and exploring the roles of MAOA in more types of immune cells should positively affect immunotherapy. Finally, ideal strategies should combine MAOAIs with chemotherapy to update drug formulations, achieve clinical translation, optimize treatment regimens, delay tumor progression, and increase patient survival rates.

## Supplementary Information

Below is the link to the electronic supplementary material.

Supplementary file1 (XLSX 22 kb)

Supplementary file2 (XLSX 47 kb)

Supplementary file3 (TIFF 412 kb)

Supplementary file4 (XLSX 7 kb)

Supplementary file5 (XLSX 24 kb)

Supplementary file6 (XLSX 117 kb)

Supplementary file7 (XLSX 25 kb)

Supplementary file8 (XLSX 13 kb)

Supplementary file9 (XLSX 26 kb)

Supplementary file10 (CSV 2 kb)

Supplementary file11 (CSV 2 kb)

Supplementary file12 (DOCX 381 kb)
