# Supplementary figures and images for: Targeting monoamine oxidase A: a strategy for inhibiting tumor growth with both immune checkpoint inhibitors and immune modulators
Source: Cancer Immunol Immunother. 2024 Feb 13;73(3):48. doi: 10.1007/s00262-023-03622-0 (PMC10864517; doi:10.1007/s00262-023-03622-0)

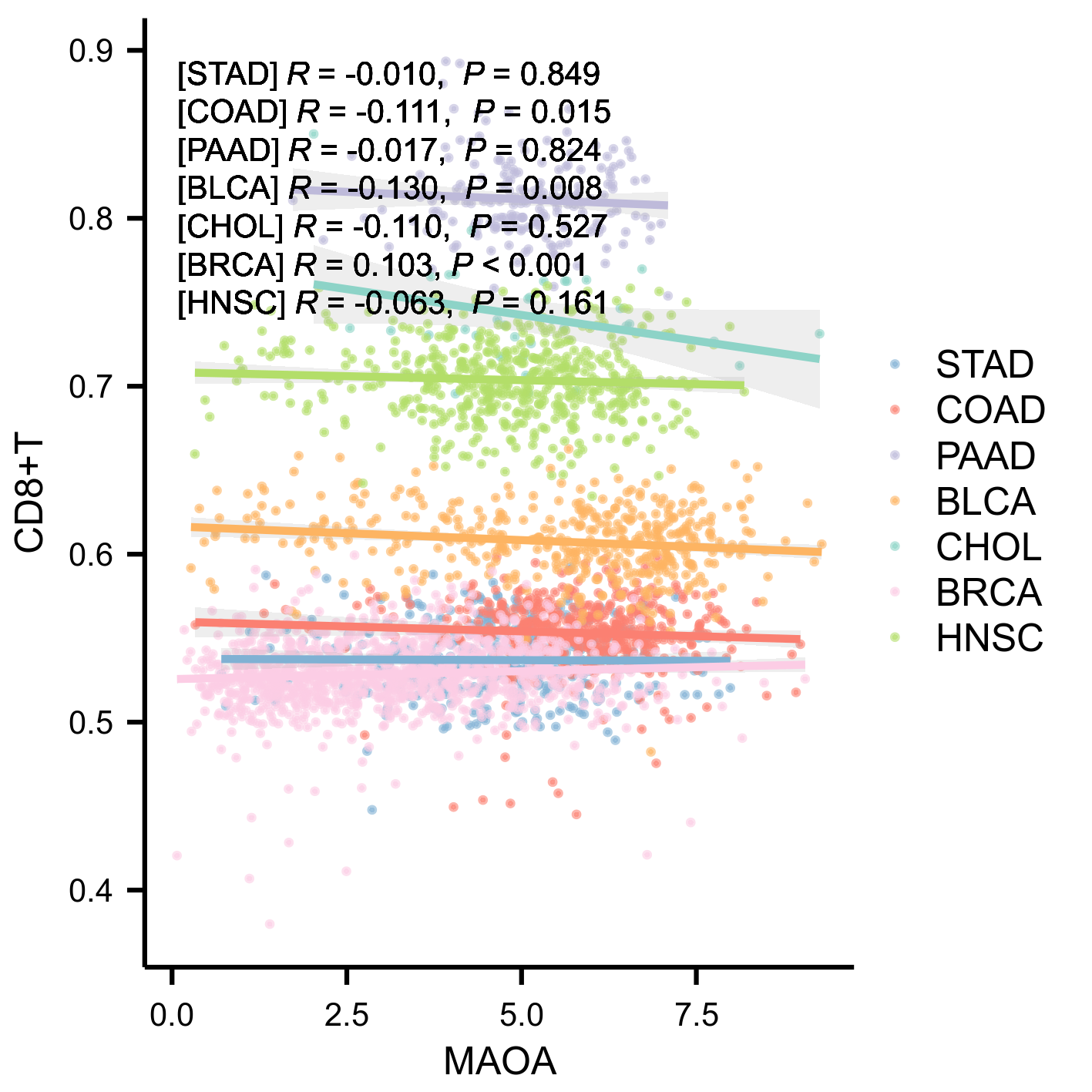

Supplement: Supplementary file 3 — Supplementary file3 (TIFF 412 kb) [file 262_2023_3622_MOESM3_ESM.tiff]

**
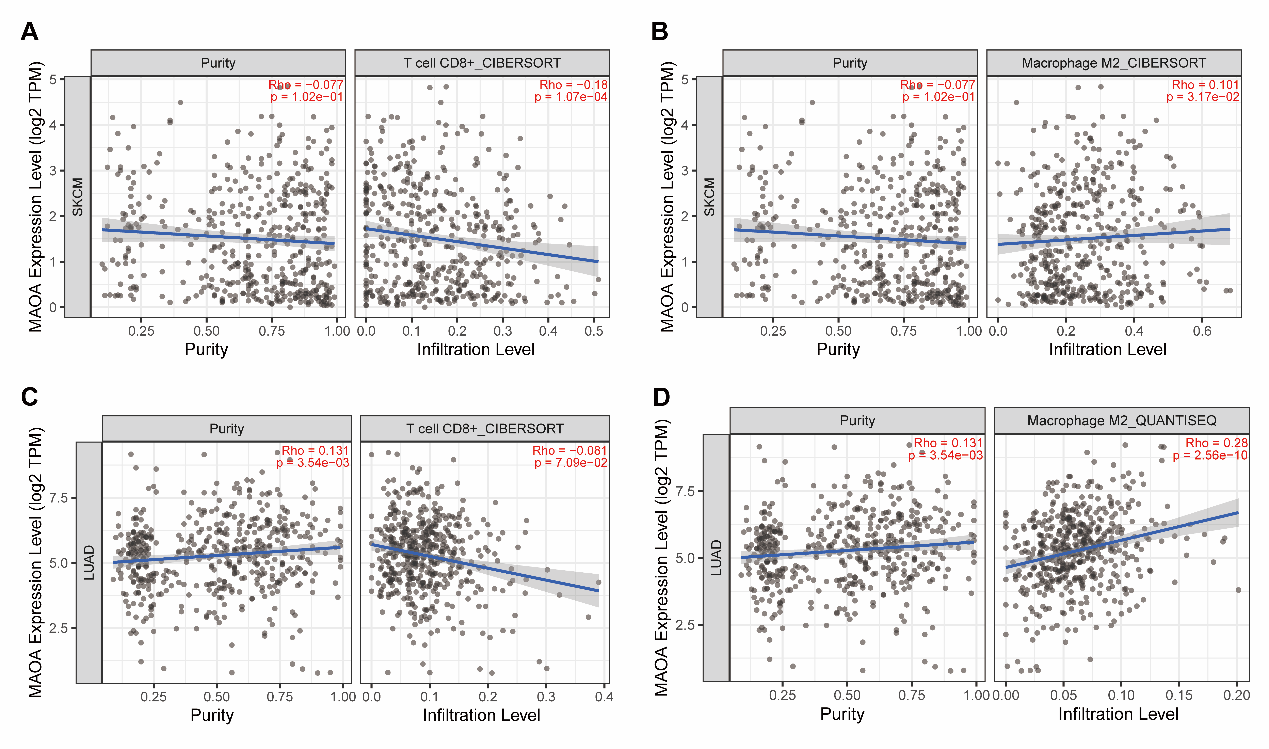
**

**Figure S1:** Immune infiltration of MAOA in melanoma and lung adenocarcinoma s

Supplement: Supplementary file 12 — Supplementary file12 (DOCX 381 kb) [file 262_2023_3622_MOESM12_ESM.docx]
